# Assessing the impact of COVID-19 on self-reported levels of depression during the pandemic relative to pre-pandemic among Canadian adults

**DOI:** 10.1186/s13690-024-01253-0

**Published:** 2024-03-06

**Authors:** Rasha Elamoshy, Marwa Farag, Nigatu Geda, Cindy Feng

**Affiliations:** 1 School of Public Health, University of Saskatchewan, Saskatoon, SK Canada; 2 College of Development Studies, Addis Ababa University, Addis Ababa, Ethiopia; 3 Department of Community Health and Epidemiology, Faculty of Medicine, University of Dalhousie, Halifax, NS Canada

**Keywords:** COVID-19, Depression, Social isolation, Financial strain

## Abstract

**Objectives:**

This study aims to assess the impact of COVID-19 related risk factors on self-reported increases in depression among Canadian adults during the pandemic compared to pre-pandemic levels. We aim to investigate the interactive effects of stressors, including social isolation, financial stress, and fear of catching COVID-19, on mental health outcomes. Our study aims to provide insights for the development of prevention and intervention strategies to address the mental health effects of the pandemic by examining the psychological changes attributable to the pandemic and its impact.

**Methods:**

This study used data collected from the Mental Health Research Canada online survey during the third wave of COVID-19 (April 20–28, 2021). The study examined the impact of COVID-19 related factors, including social isolation, financial concerns, fear of catching COVID-19, and concerns about paying bills, on self-reported increases in depression. Multivariable logistic regression models were utilized to examine these associations, with adjustments made for potential confounding variables. All statistical analysis was performed using SAS V9.4 (SAS Institute Inc., Cary, NC, USA).

**Results:**

Participants reporting social isolation, financial concerns, and fear of catching COVID-19 were more likely to report increased depression. An interaction was observed between concerns for paying bills and catching COVID-19 in relation to depression (*p* = 0.0085). In other words, the effect of concerns about paying bills on depression was stronger for individuals who also had a fear of catching COVID-19, and vice versa. Young adults, females, patients with pre-existing depression, and residents of certain provinces reported higher levels of depression during COVID-19.

**Conclusion:**

Our study underscores the significant impact of the COVID-19 pandemic on mental health, particularly among certain demographic groups. It emphasizes the need for depression screening and increased support for mental health during the pandemic, with a focus on mitigating financial burdens and reducing negative psychological impacts of social isolation. Our findings highlight the complex interplay between different stressors and the need to consider this when designing interventions to support mental health during times of crisis.

**Table:** 

**Text box 1. Contributions to the literature**
• Unlike many COVID-19 psychological impact studies, our research provides a comparative analysis of pandemic and pre-pandemic psychological changes, particularly relevant to the general Canadian adult population
• Our findings identify social isolation, financial concerns, young adulthood, female gender, and pre-existing clinically diagnosed depression as factors associated with increased depression during COVID-19
• Highlighted risk factors underscore the need for targeted interventions, widespread depression screening, and enhanced treatment strategies for emerging and worsening mental disorders
• Our research emphasizes the necessity of adaptable mental health care in response to future pandemics, highlighting the impact of public health measures on Canadians’ mental wellbeing

## Introduction

The COVID-19 pandemic caused unprecedented global disruptions, with governments and organizations implementing various measures to control the virus’s spread. Lockdowns, curfews, travel restrictions, mandatory working from home, and the suspension of non-essential health and public services were effective in reducing transmission rates, but they also createded an environment of social isolation, financial strain, fear, and uncertainty, all of which had a detrimental impact on mental health [1].

Several studies reported that social isolation led to lower levels of mental well-being [2, 3], lower quality of life [4], and higher levels of depression [5]. Financial strains were also psychologically challenging and had been associated with higher distress [6], anger, anxiety [7], and depression [8]. Additionally, fear of catching COVID-19 was another stressor that had been shown to affect mental health [9]. Moreover, COVID-19 has had a disproportionate impact on different groups [10]. Young people [10–13], women [1, 12, 14], students [14, 15], and individuals with low income [16] suffered a higher burden of mental distress during the pandemic. Those with pre-existing clinically diagnosed mental disorders, especially depression and anxiety, also experienced a significant increase in stress levels during the pandemic [17].

While numerous studies reported negative impacts of the COVID-19 pandemic on mental health, there are also studies that found no significant changes or even positive changes in mental health during the pandemic. For instance, previous research identified substantial increases in distress in the US during the emergence of the COVID-19 crisis that largely diminished in the weeks that followed [18], suggesting that population-level resilience in mental health may have occured in response to the pandemic. Similarly, a study in Norway [19] found stable levels of mental disorders, suicidal ideation, and suicide deaths during the first six months of the COVID-19 pandemic compared to pre-pandemic levels, except for a decrease in mental disorders in the first pandemic period. However, many of these studies were conducted at different time periods during the pandemic, which limits the ability to generalize their findings to other populations or time periods for studying the actual psychological changes attributable to the pandemic and its impact.

Despite the mixed findings in the literature, there is overwhelming evidence that the pandemic had a detrimental impact on mental health. As such, it is crucial to systematically examine the effects of the pandemic on mental health outcomes among Canadian adults. This study aims to contribute to this understanding by examining the impact of COVID-19 on depression levels and psychological changes relative to pre-pandemic levels among Canadian adults. In addition, we will investigate specific mitigating factors that have been identified in previous research, such as the impact of social isolation, financial stress, and fear of catching COVID-19 on mental health outcomes, including depression. While many studies examined the impact of COVID-19 on mental health, fewer explored the interactive effects of multiple stressors. Understanding how different stressors interact and exacerbate their impact on mental health during a pandemic is essential for developing targeted interventions and providing appropriate support to those in need. Unlike previous studies that focused on specific subpopulations, our study will focus on the general Canadian adult population. By doing so, we hope to provide a more comprehensive understanding of the psychological impact of COVID-19 on the broader population. Finally, our study aims to provide insights that can inform the development of effective prevention and intervention strategies to address the mental health effects of future pandemics.

## Methods

### Study sample

This study used data from Poll 6 of the National Polling on Canadian Mental Health, an ongoing series of cross-sectional surveys conducted by Pollara Strategic Insights on behalf of Mental Health Research Canada (MHRC). The survey is part of a larger effort to track the mental health challenges of COVID-19 and beyond, consisting of studies in an ongoing series. The goal of the study was to capture Canadians’ reported perception of their level of anxiety and depression and to identify and evaluate the factors that influence mental health through the pandemic.

Poll 6 was conducted from April 20 to April 28, 2021, during the third wave of the COVID-19 pandemic in Canada, when several provinces implemented strict public health measures to control the spread of the virus. This timeframe was of particular interest, as it provided a snapshot of mental health during a critical time in the pandemic. During this period, the predominant variant of COVID-19 in Canada was the B.1.1.7 variant, also known as the UK variant, which is known to be more transmissible and associated with higher hospitalization and death rates than the original strain.

The data consists of a sample of 4,005 Canadian adults collected through an online platform. The sample included an oversample of 500 surveys with residents of New Brunswick and an oversample of 500 surveys with residents of Newfoundland. The data were weighted based on age, gender, and region to be representative of the Canadian population, excluding the northern territories. While the study’s findings should be interpreted in the context of the specific time period and geographic coverage, the sample is still considered to be broadly representative of the Canadian population. Results from a probability sample of this size could be considered accurate to within ± 2.2% points [20].

### Outcome variables

The primary outcome variable was self-reported increased levels of depression due to the pandemic. Participants rated their depression levels before and since the COVID-19 outbreak in Canada on a Likert scale from 0 to 10, with 0 being “none” and 10 being “extremely high”. We calculated the difference between the two scores by subtracting the pre-pandemic score from the during-pandemic score. This allowed us to examine the impact of COVID-19 on the magnitude of psychological changes during the pandemic relative to before the pandemic. Participants with a negative difference score, indicating an improvement in their depression status since COVID-19, were excluded from the analysis. The decision to exclude individuals who reported an improvement in their depression scores (approximately 5.6% of all survey respondents) was made after careful consideration. Despite their self-reported improvement, over 50% consistently expressed concerns such as social isolation, financial worries, or fear of contracting COVID-19, indicating ongoing challenges affecting their mental well-being. This raised doubts about the reliability of their responses to the depression evaluation questions. While we acknowledge the potential variability in individual experiences with a common stressor like COVID-19, we considered this subgroup unrepresentative of those genuinely experiencing improved mental health due to inconsistencies in their responses. Furthermore, combining stabilization and improvement groups could blur the distinctions between them and potentially distort the analysis. Therefore, we opted for a more stringent approach by excluding this group. While self-reported measures of depression may have limited clinical relevance, they are commonly used in population-based research and can provide valuable insights into the subjective experiences of individuals. It is important to note that the depression levels reported by participants may not correspond to clinical diagnoses. However, this measure allowed us to assess the impact of the pandemic on self-reported changes in mood, which is a relevant outcome in the context of a global health crisis.

We derived two dichotomized outcome variables to examine the impact of COVID-19 on psychological changes during the pandemic relative to before the pandemic. To detect an increase in depression levels, we set the cutoff point at 0. To detect a higher level of depression, we set the cutoff point at the top quartile (i.e., 75th percentile) corresponding to a 2-point increase in the depression score during the pandemic relative to pre-pandemic levels. Although these specific cutoff points were not based on previous literature, we reasoned that a difference of zero would be the most conservative estimate for detecting an increase in depression levels, while the 75th percentile represented a meaningful threshold for identifying participants who experienced a higher level of increase based on our interpretation of the data distribution. We acknowledge that using a continuous difference in self-reported depression levels may provide more detailed information about changes in depression over time, but due to concerns about the validity of self-reported data and potential biases in mood-dependent rating, we chose to dichotomize the outcome variable. This allowed for a more conservative analysis that focused on detecting changes in depression levels.

### Covariates

- *Demographic characteristics*: age (18 to < 30; 30 to < 40; 40 to < 50; 50 to < 60 and 60 to < 101 years old) and gender (male vs. female).
- *Socioeconomic characteristics*: total household income in 2020 (less than $30,000; $30,000 to < $50,000; $50,000 to < $80,000; $80,000 to < $100,000; $100,000 to < $150,000; and $150,000 or more) and highest level of education completed (elementary or high school, college, technical/trade school or apprenticeship, university undergraduate degree, and university graduate/professional degree).
- *Geographical location*: Since public health restrictions varied greatly during the pandemic, the province of residence was included. Provinces in this analysis were grouped as follows: British Columbia, Alberta, Manitoba/Saskatchewan, Ontario, Quebec, Newfoundland and Labrador, New Brunswick, and the rest of the Atlantic. This grouping was mainly to improve statistical power and avoid unstable and unreliable estimates in provinces with a low number of participants. However, we tried to respect geographical proximity.
- *Past mental health conditions*: depression or anxiety disorders clinically diagnosed before COVID-19 (March 2020).
- *COVID-19 related variables*: The participant’s perception of the impact of COVID-19 related factors on their mental health is also considered. These factors included social isolation (being apart from others), the possibility of catching COVID-19, the possibility of a family member catching COVID-19, the economic downturn, and the possibility of not being able to fully pay household bills owed in 2021. Participants were asked to rate each of these factors in terms of their impact on their mental health during the current COVID-19 outbreak in Canada on a scale from 0 to 10. Responses were rated on a scale from 0 to 10 where 0 represents “a very negative” impact, 5 “neutral or no effect,” and 10 “very positive” impact. Responses from 0 to 4 were coded as “negative,” while all other responses were coded as “others.” These variables were included to assess the potential impact of COVID-19 related factors on the association between the pandemic and increased levels of depression.

### Statistical analysis

We conducted cross-tabulations to examine the distribution of participants’ characteristics by their depression status. We calculated crude unadjusted associations between different exposure variables and depression status using univariate logistic regression models. Variables with a *p*-value < 0.25 in the univariate analysis were considered for inclusion in the multivariable logistic regression models. We constructed two multivariable logistic regression models: (i) for any increase in the self-reported level of depression during the pandemic compared to before the pandemic, and (ii) for levels of self-reported depression during the pandemic that were above 2-points (75th percentile) compared to before the pandemic. We conducted backward model selection, retaining variables with *p*-values < 0.05 in the final multivariable model. We evaluated effect modifications by examining all potential two-way interactions between variables included in the main effect model. We assessed all interactions based on their *p*-value and the change in the model’s Akaike information criterion (AIC). We assessed multicollinearity among predictors using the variance inflation factor (VIF), with a cut-off of 2.5 for VIF [21].

For the multivariable model, a receiver operating characteristic (ROC) curve was generated and the area under the curve was calculated to examine model predictability. All statistical analysis was performed using SAS V9.4 (SAS Institute Inc., Cary, NC, USA). All analyses were weighted using the weighting variable provided in the data set to be representative of the study population. The SURVEYLOGISTIC command was used to adjust for the survey sampling structure. The NOMCAR option, which assumes that missing values are not missing completely at random, was used to ensure that variance estimates were accurately adjusting for the missing data.

## Results

In total, 4,005 Canadian adults participated in the survey. Of these, 208 participants did not answer questions related to their self-perceived levels of depression or depression diagnosis. Among the participants who completed the survey, 214 reported an improvement in their depression level during COVID-19 and were excluded from the analysis. The final sample used for this analysis included 3,583 participants. Overall, 1,882 (49.6%) participants reported an increase in their level of depression during COVID-19, and 810 (21.4%) participants reported an increase in depression levels of at least 2 points. Table 1 displays the distribution of the study participants’ characteristics based on their self-reported increased level of depression.

**Table 1:** Description of study participants’ characteristics stratified by increased self-reported level of depression during the third wave of COVID-19 (April 2021) versus before COVID-19 (March 2020) among adult Canadians

Variable	Category	Any self-reported increase in depression during versus before COVID-19		Above top 75th percentile of self-reported increase in depression during versus before COVID-19		
		No *N* (%)	Yes *N* (%)	No *N* (%)	Yes *N* (%)	Total *N* (%)
		1701 (47.47)	1882 (52.53)	2773 (77.39)	810 (22.61)	3583 (100)
*Socio-demographical variables*						
Age (*n* = 3583)	18 to < 30	199 (5.55)	369 (10.30)	408 (11.39)	160 (4.47)	568 (15.85)
	30 to < 40	242 (6.75)	371 (10.35)	440 (12.28)	173 (4.83)	613 (17.11)
	40 to < 50	287 (8.01)	338 (9.43)	468 (13.06)	157 (4.38)	625 (17.44)
	50 to < 60	373 (10.41)	346 (9.66)	580 (16.19)	149 (3.88)	719 (20.07)
	60 to < 101	600 (16.75)	458 (12.78)	877 (24.48)	181 (5.05)	1058 (29.53)
Gender (*n* = 3570)	Female	773 (21.65)	1029 (28.82)	1346 (37.70)	456 (12.77)	1802 (50.48)
	Male	924 (25.88)	844 (23.64)	1418 (39.72)	350 (9.80)	1768 (49.52)
*Socio-economic variables*						
Household Income (*n* = 3307)	Less than 30,000	221 (6.68)	280 (8.47)	382 (11.55)	119 (3.60)	501 (15.15)
	30,000 to < 50,000	234 (7.08)	268 (8.10)	379 (11.46)	123 (3.72)	502 (15.18)
	50,000 to < 80,000	335 (10.13)	371 (11.22)	541 (16.36))	165 (4.99)	706 (21.35)
	80,000 to < 100,000	272 (8.22)	281 (8.50)	455 (13.76)	98 (2.96)	553 (16.72)
	100,000 to < 150,000	314 (9.50)	331 (10.01)	496 (15.00)	149 (4.51)	645 (19.50)
	150,000 or more	197 (5.96)	203 (6.14)	315 (9.53)	85 (2.57)	400 (12.10)
Education (*n* = 3573)	Elementary/High school	347 (9.71)	358 (10.02)	542 (15.17)	163 (4.56)	705 (19.73)
	College	287 (8.03)	355 (9.94)	477 (13.35)	156 (4.62)	642 (17.97)
	Technical/Trade-school	292 (8.03)	273 (7.64)	455 (12.73)	110 (3.08)	565 (15.81)
	University undergraduate	483 (13.52)	586 (16.40)	828 (23.17)	241 (6.75)	1069 (29.92)
	University graduate/professional degree	286 (8.00)	306 (8.56)	463 (12.96)	129 (3.61)	592 (16.57)
*Geographic variables*						
Province (*n* = 3583)	Rest of the Atlantic	96 (2.68)	77 (2.15)	143 (3.99)	30 (0.84)	173 (4.83)
	New Brunswick	249 (6.95)	198 (5.53)	359 (10.02)	88 (2.64)	447 (12.48)
	Newfoundland and Labrador	232 (6.48)	217 (6.06)	370 (10.33)	79 (2.20)	449 (12.53)
	Quebec	293 (8.18)	260 (7.26)	441 (12.31)	112 (3.13)	553 (15.43)
	Ontario	326 (9.10)	476 (13.28)	575 (16.05)	227 (6.34)	802 (22.38)
	Manitoba/Saskatchewan	128 (3.57)	144 (4.02)	206 (5.75)	66 (1.84)	272 (7.59)
	Alberta	200 (5.58)	245 (6.84)	340 (9.49)	105 (2.93)	445 (12.42)
	British Columbia	177 (4.94)	265 (7.40)	339 (9.46)	103 (2.87)	442 (12.34)
*Past mental health conditions*						
Clinical diagnosis of depression (*n* = 3583)	No	1496 (41.75)	1476 (41.19)	2332 (65.09)	640 (17.86)	2972 (82.95)
	Yes	205 (5.72)	406 (11.33)	441 (12.31)	170 (4.74)	611 (17.05)
Clinical diagnosis of anxiety (*n* = 3565)	No	1494 (41.91)	1491 (41.82)	2344 (65.75)	641 (17.98)	2985 (83.73)
	Yes	199 (5.58)	381 (10.69)	418 (11.73)	162 (4.54)	580 (16.27)
**Self-perceived COVID-19 related factors that negatively impacted mental health**						
*Psycho-social*						
Social isolation (*n* = 3545)	Negative	940 (26.52)	1440 (40.62))	1727 (48.72)	653 (18.42)	2380 (67.14)
	Others	731 (20.62)	434 (12.24)	883 (30.07)	153 (4.32)	1165 (32.86)
*Health related*						
Possibility of catching COVID (*n* = 3503)	Negative	717 (20.47)	1054 (30.09)	1307 (37.31)	464 (13.25)	1771 (50.56)
	Others	936 (26.72)	796 (22.72)	1401 (39.99)	331 (9.45)	1732 (49.44)
Possibility of family member catching COVID (*n* = 3525)	Negative	891 (25.28)	1218 (34.55)	1594 (45.22)	515 (14.61)	2109 (59.83)
	Others	774 (21.96)	642 (18.21)	1132 (32.11)	284 (8.06)	1416 (40.17)
*Financially related*						
Economic downturn (*n* = 3512)	Negative	873 (24.86)	1163 (33.12)	1528 (43.51)	508 (14.46)	2036 (57.97)
	Others	778 (22.15)	698 (19.87)	1183 (33.68)	293 (8.34)	1476 (42.03)
Ability to pay bills (*n* = 3294)	Negative	471 (14.30)	800 (24.29)	879 (26.68)	392 (11.90)	1271 (38.59)
	Others	1082 (32.85)	941 (28.57)	1654 (50.21)	369 (11.20)	2023 (61.41)

The unadjusted analysis (Table 2) showed that compared to their counterparts, women, younger age groups, and residents of Ontario, Alberta, Saskatchewan/Manitoba, and British Columbia were more likely to report an increase in depression levels during COVID-19. Additionally, participants who had been clinically diagnosed with depression or anxiety before COVID-19 were also at higher risk. Those who reported college as the highest level of education and those who lived in Ontario had higher odds of reporting a higher increased level (> 75th percentile) of depression. Participants who reported that any of the COVID-19 related factors negatively impacted their mental health had a significantly higher risk of reporting an increase in their level of depression during versus before COVID-19.

**Table 2:** Unadjusted odds ratios for the cross-sectional association of covariates with increased self-reported level of depression during the third wave of COVID-19 (April 2021) versus before COVID-19 (March 2020) among adult Canadians

Variable	Category	Any self-reported increase in depression during versus before COVID-19			Above top 75th percentile of self-reported increase in depression since versus before COVID-19		
		Odds Ratio	95% CI		Odds ratio	95% CI	
			Lower	Upper		Lower	Upper
Age (Ref: 60 to < 101)	18 to < 30	2.1	1.67	2.75	1.88	1.42	2.49
	30 to < 40	1.85	1.45	2.35	1.88	1.42	2.48
	40 to < 50	1.36	1.07	1.73	1.55	1.16	2.06
	50 to < 60	1.03	0.82	1.29	1.11	0.83	1.48
Gender (Ref: Male)	Female	1.46	1.25	1.71	1.40	1.16	1.68
Household Income (Ref: 150,000 or more)	Less than 30,000	1.30	0.95	1.78	1.15	0.80	1.66
	30,000 to < 50,000	1.17	0.86	1.6	1.23	0.85	1.76
	50,000 to < 80,000	1.11	0.83	1.48	1.11	0.79	1.56
	80,000 to < 100,000	1.01	0.74	1.36	0.87	0.60	1.27
	100,000 to < 150,000	1.04	0.78	1.38	1.14	0.81	1.61
Education (Ref: University graduate/professional degree	Elementary/High school	1.04	0.80	1.35	1.19	0.87	1.61
	College	1.22	0.93	1.59	1.43	1.05	1.95
	Technical/Trade school	0.85	0.64	1.12	0.89	0.63	1.25
	University undergraduate	1.18	0.93	1.50	1.18	0.89	1.57
Province (Ref: New Brunswick)	Rest of the Atlantic	1.03	0.70	1.52	0.99	0.60	1.65
	Newfoundland and Labrador	1.38	0.99	1.90	1.07	0.69	1.66
	Quebec	1.15	0.88	1.52	0.97	0.69	1.37
	Ontario	1.94	1.51	2.51	1.56	1.15	2.13
	Manitoba/Saskatchewan	1.54	1.12	2.13	1.25	0.84	1.84
	Alberta	1.66	1.25	2.21	1.24	0.87	1.76
	British Columbia	1.93	1.44	2.58	1.18	0.83	1.70
Clinical diagnosis of depression (Ref: No)	Yes	2.24	1.80	2.79	1.37	1.09	1.74
Clinical diagnosis of anxiety (Ref: No)	Yes	1.93	1.54	2.42	1.39	1.10	1.77
**Self-perceived COVID-19 related factors that negatively impacted mental health**							
*Psycho-social*							
Social isolation (Ref: Others)	Negative	2.60	2.18	3.09	2.31	1.84	2.90
*Health related*							
Possibility of catching COVID (Ref: Others)	Negative	1.63	1.39	1.91	1.44	1.19	1.74
Possibility of family member catching COVID (Ref: Others)	Negative	1.62	1.38	1.91	1.25	1.03	1.51
*Financially related*							
Ability to pay bills (Ref: Others)	Negative	1.92	1.62	2.28	2.00	1.65	2.43
Economic downturn (Ref: Others)	Negative	1.52	1.29	1.79	1.29	1.06	1.56

**Table 3:** Multivariable logistic regression analysis examining the cross-sectional association of covariates with increased self-reported level of depression during the third wave of COVID-19 (April 2021) versus before COVID-19 (March 2020) (*n* = 3212) among adult Canadians

Variable	Category	Any self-reported increase in depression during versus before COVID-19	Above top 75th percentile of self-reported increase in depression since versus before COVID-19
		AOR (95%CI)	AOR (95%CI)
Age (Ref: 60 to < 101)	18 to < 30	1.79 (1.34–2.38)	1.54 (1.13–2.11)
	30 to < 40	1.57 (1.19–2.07)	1.60 (1.18–2.17)
	40 to < 50	1.30 (0.99–1.70)	1.47 (1.08–2.01)
	50 to < 60	0.93 (0.72–1.20)	1.03 (0.7–1.39)
Gender (Ref: Male)	Female	1.42 (1.19–1.69)	1.40 (1.15–1.71)
Household Income (Ref: 150,000 or more)	Less than 30,000	-	-
	30,000 to < 50,000	-	-
	50,000 to < 80,000	-	-
	80,000 to < 100,000	-	-
	100,000 to < 150,000	-	-
Education (Ref: University graduate/professional degree)	Elementary/High school	-	-
	College		
	Technical/Trade school	-	-
	University undergraduate	-	-
Province (Ref: New Brunswick)	Alberta	1.57 (1.14–2.17)	1.32 (0.78–1.4)
	British Columbia	2.01 (1.45–2.78)	1.13 (0.77–1.66)
	Manitoba/Saskatchewan	1.63 (1.14–2.34)	1.23 (0.82–1.86)
	Newfoundland and Labrador	1.62 (1.14–2.30)	1.15 (0.74–1.79)
	Ontario	2.15 (1.62–2.85)	1.59 (1.15–2.21)
	Quebec	1.18 (0.86–1.61)	0.89 (0.62–1.29)
	Rest of Atlantic	1.16 (0.76–1.79)	1.04 (0.60–1.79)
Clinical diagnosis of depression (Ref: No)	Yes	2.06 (1.60–2.66)	-
Clinical diagnosis of anxiety (Ref: No)	Yes	-	-
**Self-perceived COVID-19 related factors that negatively impacted mental health**			
*Psycho-social*			
Social isolation (Ref: Others)	Negative	2.34 (1.93–2.85)	2.03 (1.59–2.59)
*Health related*			
The possibility of catching COVID (Ref: Others)	Negative	1.31 (1.10–1.57)	-
Possibility of a family member catching COVID (Ref: Others)	Negative	-	-
*Financially related*			
Concern on paying bills (Ref: Others)	Negative	1.44 (1.19–1.74)	1.66 (1.35–2.04)
Economic downturn (Ref: Others)	Negative	-	-

Table 3 presents the results of the multivariable analysis. Females were found to be 1.42 times more likely than males to report an increase in their level of depression during COVID-19 (AOR = 1.42, 95% CI: 1.19–1.69). Participants aged 18 to < 30 (AOR = 1.79, 95% CI: 1.34–2.38) and 30 to < 40 years old (AOR = 1.57, 95% CI: 1.19–2.07) had a higher risk of reporting an increase in depression compared to those aged 60 years or older. This pattern was also observed among participants who reported a higher increase in their level of depression. Moreover, the odds of those aged 40 to 50 years reporting above the 75th percentile in the increased level of depression were 47% higher than the odds of those older than 60 (AOR = 1.47, 95% CI: 1.08–2.01).

Residents of Alberta, British Columbia, Manitoba/Saskatchewan, and Newfoundland and Labrador were significantly more likely to report an increase in their level of depression during COVID-19 compared to the residents from New Brunswick. No significant differences were found from the rest of Atlantic Canada and New Brunswick. Only residents of Ontario had significantly higher odds of reporting both any increase (AOR = 2.15, 95% CI:1.62–2.85) and > 75th percentile increase in the level of depression (AOR = 1.59, 95% CI: 1.15–2.21). Patients who were clinically diagnosed with depression were twice as likely to report an increase in their level of depression during COVID-19 compared to those who were not diagnosed with the condition (AOR = 2.06, 95% CI: 1.60–2.66), suggesting that the psychological impacts of the pandemic may be more concerning for this subgroup.

Regarding COVID-19-related factors, social isolation was the strongest predictor of elevated depression during the pandemic. Participants who believed social isolation had a negative impact on their mental wellbeing were twice as likely to report increased levels of depression (AOR = 2.34, 95% CI: 1.93–2.85, any increase, and AOR = 2.03, 95% CI: 1.59–2.59, > 75th percentile) compared to those who did not believe so. Similarly, individuals who were concerned about their ability to pay bills and its impact on their mental health were more likely to report increased depression (AOR = 1.44, 95% CI: 1.19–1.74, any increase, and AOR = 1.66, 95% CI: 1.35–2.04, > 75th percentile). However, being concerned about the possibility of a family member catching COVID-19 and the economic downturn did not significantly affect the likelihood of reporting increased levels of depression during COVID-19. The area under the receiver operating characteristic (ROC) curve for the final model was approximately 70%.

**Fig. 1:**
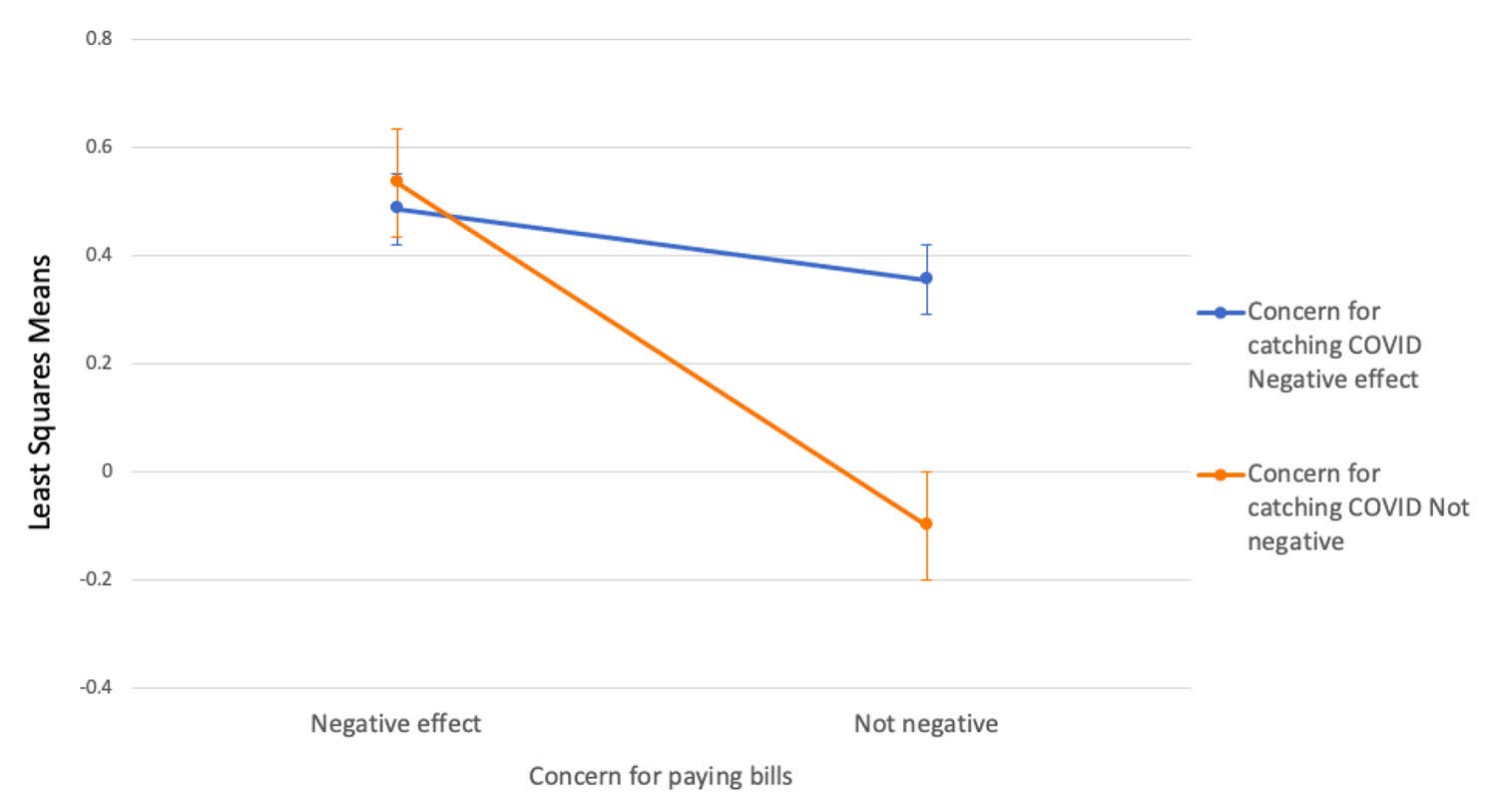
Least squares means (estimates of the linear predictors on the logit scale) and the 95% confidence intervals of increased depression for those who believed that their concerns for paying bills negatively impacted their mental health and those who did not, stratified by whether they expressed concerns about catching COVID-19. Cross-sectional examination of increased self-reported depression among adult Canadians during the third wave of COVID-19 (April 2021) versus before COVID-19 (March 2020)

A significant interaction was observed between the variables of concern for paying bills and concern for catching COVID-19 with respect to any increase in depression levels during the pandemic (*p*-value = 0.0085). As shown in Fig. 1, among individuals who reported no concerns about paying bills, those who were also concerned about catching COVID-19 had 1.58 times higher odds of reporting increased depression compared to those who had no concerns about catching the virus (AOR = 1.58, 95% CI: 1.26–1.98). However, for individuals who were concerned about paying bills, there was no significant difference in the odds of increased depression between those who were concerned about catching COVID-19 and those who were not concerned (AOR = 0.95, 95% CI: 0.71–1.29).

## Discussion

This study adds to the growing body of literature examining the impact of COVID-19 on mental health. Although several studies have explored the prevalence of depression during the pandemic, our study provides a unique contribution by examining the change in depression levels from before the pandemic to during the third wave in a Canadian context.

Our findings show a high prevalence of increased self-reported depression levels, with nearly half of the participants reporting an increase and over one-fifth reporting a substantial increase. These results are consistent with other studies that have used standardized depression screening tools. For example, a recent meta-analysis of 12 studies showed that the prevalence of depression during COVID-19 ranged from 18 to 33%, with a pooled estimate of 25%, compared to 3.44% prior to COVID-19 [22]. Other systematic reviews also showed similar trends among different populations [23, 24]. In Canada, a study evaluated the prevalence of major depressive disorders during COVID-19 and compared this to pre-COVID-19 estimated rates, reporting an increase from 7% before the pandemic to more than 13% during the pandemic [25]. However, there are also studies that have found no significant changes or even positive changes in mental health during the pandemic. For instance, a study in Norway found stable levels of mental disorders, suicidal ideation, and suicide deaths during the first six months of the COVID-19 pandemic compared to pre-pandemic levels, except for a decrease in mental disorders in the first pandemic period [19]. Overall, the inconsistency in the literature highlights the need for continued research to better understand the impact of the pandemic on mental health and to identify factors that may promote resilience and positive mental health outcomes.

Our results showed that perceived social isolation was the strongest and most consistent predictor of increased depression. Previous studies have shown that even short periods of isolation are linked to acute stress disorders [26], post-traumatic stress disorders [27], and depressive symptoms [28]. These psychological effects were still detected even three years later [27]. Furthermore, our study participants who had uncertainty about their ability to pay bills had a higher risk of experiencing increased depression after COVID-19. This finding is consistent with other [29]. Financial strain during a pandemic is usually due to pay cuts and financial loss without prior preparedness, representing a major threat to mental wellbeing. Prior research has shown that financial stress is associated with higher psychological distress, anxiety, and depression, irrespective of income [29, 30].

A common underlying factor that may explain the effects of both social isolation and financial strains on depression is uncertainty and intolerance to uncertainty. Individuals want to fully understand the extent of the threats they face and need to feel in control. Fear of the unknown and loss of control, since even experts cannot predict what comes next, is distressing. This can cause a sense of negativity that is detrimental to mental wellbeing. Our study also showed that fear of contracting COVID-19 was significantly associated with increased levels of depression. This implies that the intense fear of catching COVID-19 may cause a physiological response.

The results of our study demonstrate a significant interactive effect between fear of catching COVID-19 and financial constraint due to the pandemic on mental health outcomes. Our findings emphasize the potential for stressors to interact and exacerbate their impact on mental health during a pandemic. Specifically, fear of catching COVID-19 may increase anxiety and stress levels, which can be further compounded by financial constraint due to job loss or reduced income. Individuals who are already experiencing financial strain may also worry about the potential financial impact of contracting the virus, such as medical bills or lost income from time off work. These findings highlight the need for interventions that consider the potential interplay between different stressors to improve mental health during a pandemic. Further research is necessary to better understand the complex interplay between different stressors and their impact on mental health outcomes during a pandemic. Future studies should explore the potential for additional stressors, such as social isolation and caregiver burden, to interact with fear of catching COVID-19 and financial constraint. Understanding the interactions between different stressors could inform the development of more targeted interventions to support mental health during times of crisis.

Consistent with previous research [18, 31–33], our study found that younger age groups were at higher risk of reporting increased levels of depression during the pandemic compared to pre-pandemic levels. This is not surprising, as young adults, especially college students, undergo stressful normative changes related to their professional, financial, and social lives [34]. These stressors were intensified by COVID-related pressures, especially social isolation and financial uncertainty. While older adults are at higher risk of suffering from adverse physical effects after a COVID-19 infection, they tend to be protected from the detrimental psychological effects of COVID stressors. This may be due to the fact that older adults have had more time to develop lower stress reactivity, better emotional regulation, and stronger psychological resilience compared to young adults [35]. Therefore, they are less likely to report an increase in depression with COVID-19.

The current study revealed that females are at a greater risk of reporting an increased level of depression, partly due to increased biological, social, and cultural vulnerability. This finding may be due to females’ socially constructed caregiving role, including caring for and supporting children and the elderly, resulting in work overload during the pandemic. Greater psychological distress in females may also result from a higher percentage of the female workforce being negatively affected by COVID-19, such as in healthcare, retail, and service industries [10]. Other research suggests that females exhibit differential neurobiological responses when exposed to stressors, resulting in a higher overall rate of mental health conditions among women [36].

The study also showed that patients with pre-existing depressive symptoms were at a higher risk of reporting increased depression during COVID-19. This finding is consistent with other studies [10, 17]. Patients with a history of mental disorders may generally be more sensitive to external stressors, such as the social isolation associated with the pandemic [10]. Additionally, hospital care was affected during the pandemic, resulting in growing backlogs or delays in hospital procedures. This may have unintentionally negatively impacted care for people with pre-existing mental health conditions. This finding highlights the vulnerability and unmet needs of patients with pre-existing mental health disorders, especially in a context where access to mental health services was disrupted, as during the COVID-19 pandemic.

In this study, the association between the province of residence and increased depression during COVID-19 was examined. The results indicate that residents of Ontario were significantly more likely to report higher levels of depression during COVID-19 compared to residents of other provinces. In contrast, residents of Quebec, New Brunswick, Nova Scotia, and Prince Edward Island reported less of an increase in depression relative to other provinces. These findings are consistent with previous research that has examined psychological distress during the pandemic and demonstrated similar patterns [33]. Additionally, pre-pandemic reports have shown that Quebec residents tend to report better mental health compared to other provinces, while Ontarians generally struggle with a higher burden of mental distress [37]. It is worth noting that Ontario is one of the most populous provinces and was hit hardest by the pandemic, reporting higher case counts and deaths compared to other provinces [38]. Therefore, the higher depression levels reported by Ontario residents may be due to the greater impact of the pandemic on their daily lives. Furthermore, the pandemic, along with variations in provincial policies to control its spread, may have highlighted pre-existing variations in mental health between provinces, as has been observed with women’s mental health, as discussed previously.

This study had several strengths, including: (1) limited potential for selection bias as participants were not recruited based on their depression status; (2) the same participant rated their level of depression before and during COVID-19, providing more consistent information; and (3) the use of statistical weighting to account for the survey sampling structure. However, this study also has some limitations that should be considered when interpreting the results, including: (1) potential recall bias as participants may have over or underestimated their level of depression prior to COVID-19; (2) self-reported evaluation of depression status; and (3) being a cross-sectional study, which precludes conclusions regarding causality. Additionally, it is worth noting that psychological responses to the pandemic may have been more prevalent in the earlier stages and gradually attenuated with time for some individuals, while for others, it may sustain over longer periods, contributing to the development of persistent mental health disorders. Future longitudinal research is necessary to determine the temporal dynamics of psychological responses to the pandemic.

### Policy implications

It is necessary for government officials to shift their mindset away from uniform approaches and recognize the varying levels of risk for different subgroups that may contribute to differences in depression and tailor their responses accordingly. Our results highlight the need for improving access to mental health services, targeted intervention, and wider depression screening during pandemics or other public health crises. Policymakers need to consider the impact of public health measures, taken to control the spread of pandemics, on Canadians’ mental wellbeing. Financial support could be provided to people who are experiencing financial hardship or loss of jobs due to the pandemic. Our findings also suggest the importance of expanding depression screening in primary care settings and providing more frequent evaluations of the needs of patients with pre-existing mental disorders. These steps can help to alleviate the significant burden of depression caused by the COVID-19 pandemic.

## Conclusion

Our study sheds light on the significant prevalence of self-reported increased depression among Canadian adults during the third wave of COVID-19. Our findings reveal that individuals who experienced social isolation, financial stress, and fear of contracting COVID-19 were more likely to report an increased level of depression during the pandemic compared to before it. Our results also suggest that the interactive effect between concern for paying bills and concern for catching COVID-19 plays a significant role in depression levels during the pandemic.

Furthermore, our study highlights several groups that are particularly vulnerable to increased depression during COVID-19, including young adults, females, residents of certain provinces (especially Ontario), and those with pre-existing clinically diagnosed depression. These findings underscore the urgent need for policymakers to develop national mental health strategies that prioritize safe and equitable access to mental health services and financial support programs for vulnerable populations.

## Data Availability

The datasets used and/or analysed during the current study is available from the corresponding author on reasonable request.
